# The undesirable communication: A case of cerebral air emboli in the setting of atrio-esophageal fistula following atrial fibrillation catheter ablation

**DOI:** 10.21542/gcsp.2022.5

**Published:** 2022-06-30

**Authors:** Momin Islam, Hussam Al Hennawi, Anwar Khedr, Khaled Harmouch, Mohammad F. Mathbout

**Affiliations:** 1 Houston Methodist Hospital, Department of Cardiology, USA; 2 Department of Internal Medicine, Jefferson Abington Hospital, Abington, PA, USA; 3 Department of Internal Medicine, BronxCare Health System, NY, USA; 4 University of Nebraska Medical Center, Omaha, Nebraska, USA; 5 Medical University of South Carolina, Department of Cardiology; Charleston, South Carolina, USA

## Abstract

Atrial-esophageal fistula is an extremely rare condition but is often a deleterious complication following catheter ablation for atrial fibrillation. The associated iatrogenic communication acts as a conduit for air and bacterial translocation, which may lead to cerebral air embolism and polymicrobial sepsis, respectively. Coupled with a history of invasive procedures, the diagnosis is largely based on the accompanying neurological symptoms. In this report, we present the case of a 73-year-old female who presented with neurological deficits attributed to cerebral vascular emboli three weeks after catheter ablation for the treatment of chronic atrial fibrillation.

## Background

Left atrial-esophageal fistula (AEF) is an extremely rare complication found in 0.03−0.08 percent of patients who undergo cardiac ablation for treatment of atrial fibrillation^1–3^. However, it is often fatal and can manifest with neurological symptoms (owing to air emboli blocking the brain vasculature) or sepsis. AEF should be considered in patients presenting with neurological deficits and sepsis several weeks after radiofrequency ablation^3^. In this report, we present the case of a 73-year-old woman who presented with neurological deficits attributed to brain emboli three weeks after catheter ablation for the treatment of refractory atrial fibrillation.

## Case presentation

A 73-year-old female patient was admitted to the hospital due to unconsciousness and deteriorating neurological manifestations. Three weeks before admission, the patient underwent radiofrequency cardiac ablation as a treatment for refractory atrial fibrillation. Following the procedure, the patient was stable, alert, and independent in all activities of daily living. On Initial evaluation in an outside hospital, her Glasgow coma scale was 9–10 with intermittent eye-opening but consistent withdrawal to painful stimuli. However, upon arrival at the hospital, she was unconscious and unresponsive to painful stimuli. Her Glasgow Coma Scale score dropped to 3, after which she was transferred and intubated in the neurological intensive care unit. The results of a general examination were unremarkable. Blood workup revealed a leukocyte count of 6.49*109/L, hemoglobin 14.2 g/L, hematocrit of 42.1%, and an elevated anion gap.

**Figure 1.**
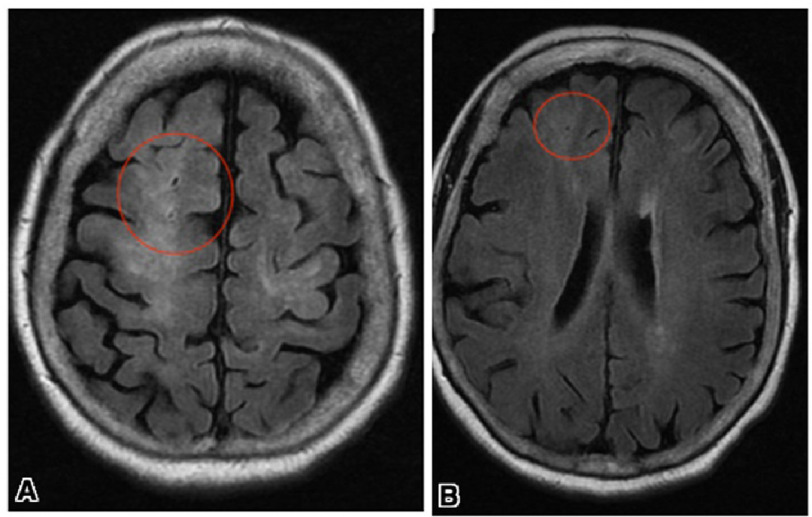
Initial brain MRI showing multifocal areas of ischemia consistent with a central embolic source in a multivessel peripheral distribution, with no evidence of hemorrhage (A). A few foci of air in the sulci within the vessels are evident (A), (B). The etiology of the multiple air emboli was unclear but the air appears to be within the bridging veins in the subarachnoid space.

Magnetic resonance imaging (MRI) of her brain was obtained delineating central air emboli in a multivessel peripheral distribution associated with multiple bilateral cerebral infarcts (Figure 1) with no evidence of hemorrhage. As a result, no neurosurgical interventions were planned, however, permissive hypertension and holding off the ventilator were suggested to prevent any possible hemorrhagic conversion.

**Figure 2.**
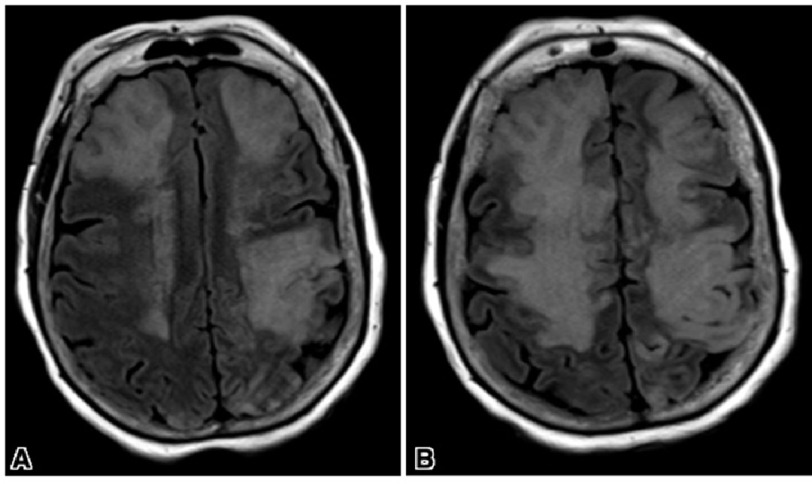
Follow-up brain MRI on day 3 showing the extensive progression of multiple areas of acute ischemic changes with the possibility of superimposed septic emboli with hemorrhage (A) with leptomeningeal involvement (B).

On the third day following admission, the patient developed fever. Blood samples were collected for culture, and the patient was started empirically on vancomycin and meropenem. Given the lack of improvement in her status, repeated MRI of the head was performed, which indicated rapid progression of acute ischemic changes in multiple areas of the brain, along with meningeal enhancement (Figure 2).

**Figure 3.**
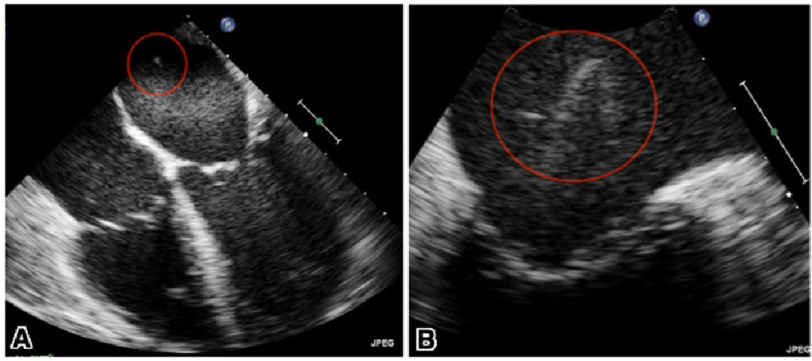
Transesophageal echocardiography showing a long, filamentous, highly mobile mass attached to the posterior wall of the left atrium (A). Magnified image demonstrating the mass measuring 1.7 cm length and 2 mm width (B).

Blood cultures were positive for *Streptococcus salivarius* and *Candida glabrata*. The polymicrobial nature of the infection suggested a plausible connection between the cardiovascular and gastrointestinal systems. A pan-CT scan was ordered to investigate for possible sepsis and locate the site of connection; however, it failed to show any abdominal pathology to account for the positive blood cultures. Subsequently, transesophageal echocardiography (TEE) was performed, which revealed a 1.7 cm ×2 mm mobile vegetation attached to the posterior wall of the left atrium (Figure 3). Our patient’s clinical condition and imaging studies led to the diagnosis of an atrioesophageal fistula (AEF), a complication of her cardiac ablation procedure, which led to vegetation formation and seeding of septic emboli. Additionally, the AEF allowed the passage of air emboli to the brain leading to pneumocephalus and multiple ischemic infarcts.

On the fifth day, the patient developed septic shock and required norepinephrine pressor support. There was no improvement in her overall clinical status, and she died on day six of her hospital admission.

## Discussion

Catheter ablation is commonly used to treat and prevent recurrence of symptomatic AF. When anti-arrhythmic medicines fail or are not tolerated in individuals with symptomatic paroxysmal, chronic, or long-standing persistent atrial fibrillation, they are used as a second-line treatment^4^. The choice to follow this treatment strategy is influenced by several criteria, including the type of AF, intensity of symptoms, presence of structural heart disease, patient preferences, and the probability of complications. Before undergoing catheter ablation, all patients should be evaluated for the possibility of adverse outcomes^5^.

Complications of catheter ablation can be classified into minor and major complications^6^, as shown in (Table 1). Various studies have reported different incidences of complications post-catheter ablation for AF. In the Nationwide Inpatient Sample analysis from 2000 to 2010 in the USA, the complication rate was 6.29%^7^. In one systematic review and meta-analysis of 192 published studies between 2000 and 2012, the overall rate of complications was 2.9%^1^. In an updated registry for procedural safety of catheter ablation for AF, the overall rate of complications was 4%, with vascular access site complications being the most common^8^.

**Table 1:** Complications of catheter ablation.

Minor complications	Major complications
Pericarditis (0–50%)	Cardiac perforation and tamponade (0.2–5%)
Vascular site access complications (0.2–1.5%)	Stroke or TIA (0–2%)
	Pulmonary venous stenosis (1–3%)
	Phrenic nerve paralysis (0–0.4%)
	Atrioesophageal fistula (0.02–0.11%)
	Myocardial infarction (<0.1%)
	Death (<0.1–0.4%)

Our patient developed AEF approximately three weeks following her catheter ablation. AEF is described as a communication between the atrium and the esophageal lumen. It often occurs 1–4 weeks after the procedure but can occur at any time beyond a 1-month duration^9,10^ and has a high mortality rate ranging from 67% to 100%^11,12^. The clinical presentation is usually subtle in the beginning, but patients can develop sudden neurologic symptoms due to esophageal air embolism^10^, consistent with our patient’s symptomatology. AEF can also present with acute inferior myocardial ischemia, heart block^9^, fever, seizures, transient ischemic attack (TIA) following food ingestion, hematemesis, and endocarditis^10^.

In our case, the translocation of bacteria, such as *Streptococcus salivarius* and *Candida glabrata,* from the gastrointestinal tract to the cardiovascular system led to positive blood culture, sepsis, vegetation formation over the left atrium, and seeding of septic emboli. Such a translocation should always raise suspicion for the possibility of a gastrointestinal source and a possible connection with the cardiovascular system. AEF is diagnosed using chest MRI or CT scan with water-soluble contrast^10^, while endoscopy with air insufflation should generally be avoided^9^. Brain imaging using MRI should also be considered in cases of neurological symptoms to diagnose cerebral emboli^9^.

However, the mechanism by which AEF occurs remains unclear. In AEF, the esophagus shows signs of thermal damage upon pathological examination. It has been hypothesized that thermal injury triggers an inflammatory response, which results in secondary perforation weeks after the procedure^10^. Therefore, the main preventive measures are lowering the catheter ablation energy settings, monitoring of esophageal temperature during the procedure, and the use of esophageal cooling devices^10^. Other preventive measures include thermal insulation of the esophagus using a balloon catheter between the left atrium and esophagus, and gastric acid suppression using high-dose proton pump inhibitor therapy^13^. Surgical repair with wide excision of the damaged segment of the esophagus followed by gastroesophageal reconstruction is the primary therapeutic option for AEF^10^.

In conclusion, AEF may have a delayed presentation after catheter ablation for atrial fibrillation. We encourage a high level of clinical suspicion in patients presenting with chest discomfort, neurologic manifestations related to new-onset stroke, gastrointestinal bleeding, and fulminant sepsis for several months following ablation. A multidisciplinary team approach may provide opportunities for better outcomes.

### What we have learned

- The estimated incidence of AEF following cardiac ablation is less than 0.1% but is associated with 67–100% mortality risk.
- Variable time of clinical presentation may lead to delayed diagnosis of underlying AEF.
- A high level of clinical suspicion should be considered in patients presenting with chest discomfort, neurological manifestations related to new-onset stroke, gastrointestinal bleeding, and fulminant sepsis for several months following ablation.
- A multidisciplinary approach may provide opportunities for better outcomes.

## Competing interests

The authors have no competing interests to declare.

## References

[1] Gupta A, Perera T, Ganesan A, Sullivan T, Lau DH, Roberts-Thomson KC, Brooks AG, Sanders P (2013). Complications of catheter ablation of atrial fibrillation: a systematic review. Circ Arrhythm Electrophysiol..

[2] Nair KK, Shurrab M, Skanes A, Danon A, Birnie D, Morillo C, Chauhan V, Mangat I, Ayala-Paredes F, Champagne J, Nault I, Tang A, Verma A, Lashevsky I, Singh SM, Crystal E (2014). The prevalence and risk factors for atrioesophageal fistula after percutaneous radiofrequency catheter ablation for atrial fibrillation: the Canadian experience. J Interv Card Electrophysiol..

[3] Han HC, Ha FJ, Sanders P, Spencer R, Teh AW, O’Donnell D, Farouque O, Lim HS (2017). Atrioesophageal fistula: clinical presentation, procedural characteristics, diagnostic investigations, and treatment outcomes. Circ Arrhythm Electrophysiol..

[4] Kirchhof P, Benussi S, Kotecha D, Ahlsson A, Atar D, Casadei B, Castella M, Diener HC, Heidbuchel H, Hendriks J, Hindricks G, Manolis AS, Oldgren J, Popescu BA, Schotten U, Van Putte B, Vardas P, Agewall S, Camm J, Baron Esquivias G, Budts W, Carerj S, Casselman F, Coca A, De Caterina R, Deftereos S, Dobrev D, Ferro JM, Filippatos G, Fitzsimons D, Gorenek B, Guenoun M, Hohnloser SH, Kolh P, Lip GY, Manolis A, McMurray J, Ponikowski P, Rosenhek R, Ruschitzka F, Savelieva I, Sharma S, Suwalski P, Tamargo JL, Taylor CJ, Van Gelder IC, Voors AA, Windecker S, Zamorano JL, Zeppenfeld K (2016). 2016 ESC Guidelines for the management of atrial fibrillation developed in collaboration with EACTS. Europace.

[5] January CT, Wann LS, Alpert JS, Calkins H, Cigarroa JE, Cleveland JC Jr, Conti JB, Ellinor PT, Ezekowitz MD, Field ME, Murray KT, Sacco RL, Stevenson WG, Tchou PJ, Tracy CM, Yancy CW ACC/AHA Task Force Members.

[6] Mahankali Sridhar AR, Wazni O, Hussein AA (2018). Ablation of atrial fibrillation: facts for the referring physician. Cleve Clin J Med..

[7] Deshmukh A, Patel NJ, Pant S, Shah N, Chothani A, Mehta K, Grover P, Singh V, Vallurupalli S, Savani GT, Badheka A, Tuliani T, Dabhadkar K, Dibu G, Reddy YM, Sewani A, Kowalski M, Mitrani R, Paydak H, Viles-Gonzalez JF (2013). In-hospital complications associated with catheter ablation of atrial fibrillation in the United States between 2000 and 2010: analysis of 93 801 procedures. Circulation.

[8] Bertaglia E, Stabile G, Pappone A, Themistoclakis S, Tondo C, De Sanctis V, Soldati E, Tritto M, Solimene F, Grimaldi M, Zoppo F, Pandozi C, Augello G, Calò L, Pappone C (2013). Updated national multicenter registry on procedural safety of catheter ablation for atrial fibrillation. J Cardiovasc Electrophysiol..

[9] Calkins H, Hindricks G, Cappato R, Kim YH, Saad EB, Aguinaga L, Akar JG, Badhwar V, Brugada J, Camm J, Chen PS, Chen SA, Chung MK, Nielsen JC, Curtis AB, Davies DW, Day JD, d’Avila A, de Groot NMSN, Di Biase L, Duytschaever M, Edgerton JR, Ellenbogen KA, Ellinor PT, Ernst S, Fenelon G, Gerstenfeld EP, Haines DE, Haissaguerre M, Helm RH, Hylek E, Jackman WM, Jalife J, Kalman JM, Kautzner J, Kottkamp H, Kuck KH, Kumagai K, Lee R, Lewalter T, Lindsay BD, Macle L, Mansour M, Marchlinski FE, Michaud GF, Nakagawa H, Natale A, Nattel S, Okumura K, Packer D, Pokushalov E, Reynolds MR, Sanders P, Scanavacca M, Schilling R, Tondo C, Tsao HM, Verma A, Wilber DJ, Yamane T (2017). 2017 HRS/EHRA/ECAS/APHRS/SOLAECE expert consensus statement on catheter and surgical ablation of atrial fibrillation: executive summary. J Arrhythm..

[10] Maan A, Shaikh AY, Mansour M, Ruskin JN, Heist EK (2011). Complications from catheter ablation of atrial fibrillation: a systematic review. Crit Pathw Cardiol..

[11] Cummings JE, Schweikert RA, Saliba WI, Burkhardt JD, Kilikaslan F, Saad E, Natale A (2006). Brief communication: atrial-esophageal fistulas after radiofrequency ablation. Ann Intern Med..

[12] Siegel MO, Parenti DM, Simon GL (2010). Atrial-esophageal fistula after atrial radiofrequency catheter ablation. Clin Infect Dis..

[13] Pappone C, Vicedomini G, Santinelli V (2013). Atrio-esophageal fistula after AF ablation: pathophysiology prevention & treatment. J Atr Fibrillation..

